# The effect of evolocumab alone and in combination with atorvastatin on atherosclerosis progression and TLRs expression

**DOI:** 10.25122/jml-2021-0210

**Published:** 2023-05

**Authors:** Ali Saud, Nabeel Ali, Fadhil Gali, Heider Qassam, Najah Rayish Hadi

**Affiliations:** 1. Department of Pharmacology and Therapeutics, College of Medicine, University of Kufa, Najaf, Iraq; 2. Department of Pharmacology, College of Medicine, University of Basra, Basra, Iraq; 3. Department of Pharmacology &Therapeutics, Faculty of Medicine, University of Kufa, Najaf, Iraq

**Keywords:** atherosclerosis, TLRs, evolocumab, atorvastatin, PCSK9: Proprotein Convertase Subtilisin/Kexin 9, CSK: Preprotein Convertase Subtilisin/Kexin 9 (alternative abbreviation), TLRs: Toll-like receptors, ICAM: Intracellular Adhesion Molecule, VCAM: Vascular Adhesion Molecule, IL: Interleukin, LDL-C: Low-Density Lipoprotein-Cholesterol, TC: Total Cholesterol, TG: Triglycerides

## Abstract

Evolocumab, a PCSK-9 inhibitor, is known for its ability to reduce low-density lipoprotein cholesterol (LDL-C). This study aimed to investigate the effects of evolocumab, alone or in combination with atorvastatin, on the progression of atherosclerosis. Fifty male domestic rabbits were randomly assigned to five groups: control, high cholesterol diet, evolocumab vehicle (dimethyl sulfoxide, DMSO), evolocumab alone, and evolocumab plus atorvastatin. Serum levels of interleukin 10 (IL-10), IL-17, IL-1β, intracellular adhesion molecule (ICAM), and vascular adhesion molecule (VCAM) were measured. Toll-like receptor (TLR) expression on monocytes was evaluated using flow cytometry. Histopathological examination and measurement of intimal thickness (IT) were also conducted. The results revealed that the evolocumab produced a statistically significant (p<0.05) reduction in lipid profile at 5 weeks, with the peak effect occurring at 10 weeks. Furthermore, the inhibitor reduced TLRs at 10 weeks to 10.83±1.8 and intimal thickness to 160.66±9.45. IL-17, IL-1β, ICAM, and VCAM were significantly reduced by evolocumab treatment, with the improvement of the histopathological changes in the aortic wall. The combination of evolocumab and atorvastatin caused a more statistically significant reduction in TLRs at 10 weeks to 5.08±1.2 and intimal thickness to 121.79±5.3. IL-17, IL-1β, ICAM, and VCAM were significantly (p<0.05) reduced by the combination, and the histopathological changes in the aortic wall were significantly improved. In conclusion, evolocumab delays the progression of atherosclerosis by modulating inflammatory pathways.

## INTRODUCTION

ICholesterol hemostasis is regulated by many protease serine enzymes. Among these enzymes, proprotein convertase subtilisin kexin 9 (PCSK9) has emerged as a key player in cholesterol metabolism [1]. Evolocumab is a well-known inhibitor interfering with the recycling of low-density lipoprotein receptors (LDL-R) at the plasma membrane of hepatocytes. It is commonly used as a monotherapy or in combination with statins to effectively reduce levels of low-density lipoprotein-cholesterol (LDL-C) [2]. Statins, the current first-line treatment for hypercholesterolemia, have shown promising results in managing atherosclerosis [3].

Atherosclerosis, characterized by chronic inflammation of blood vessels, results from an imbalance in cholesterol metabolism, leading to intracellular accumulation of low-density lipoprotein (LDL-C). These events can trigger immune responses. In 2017 alone, cardiovascular diseases caused approximately 17.79 million deaths worldwide, underscoring the urgent need to address this compelling health concern [4]. However, the precise relationship between evolocumab and inflammation in the context of atherosclerosis remains unclear.

Numerous risk factors, including hyperlipidemia [3], heavy smoking [5], uncontrolled hypertension [6], male gender [7], diabetes mellitus [8], and oxidative stress [9,10], have been implicated in the development of atherosclerosis. Toll-like receptors (TLRs) are a family of receptors involved in various inflammatory processes. Toll-like receptor 4 (TLR4), an integral membrane receptor, has been closely associated with the pathogenesis of atherosclerosis and exhibits elevated expression levels in atherosclerotic lesions at different stages of atherogenesis in both humans and murine models [11]. Recent evidence suggests that lowering low-density lipoprotein-cholesterol (LDL-C) to very low levels through evolocumab administration may lead to the regression of coronary atherosclerotic plaques in patients already receiving statin therapy [12]. Therefore, the aim of this study was to investigate the effects of evolocumab, both as a monotherapy and in combination with atorvastatin, on the progression of atherosclerosis.

## MATERIAL AND METHODS

### Experimental animals

Fifty male rabbits aged 6-12 months, weighing 1-1.5 kg, were included in this study. The rabbits were housed under standard laboratory conditions with ad libitum access to a pellet diet. The rabbits were categorized into five groups: Group I served as the normal control group and received standard chew food and water. Group II was fed a high-cholesterol diet containing 2% cholesterol. Group III received a high-cholesterol diet and was treated with dimethyl sulfoxide (DMSO) orally as a vehicle. Group IV received a high-cholesterol diet and was treated with evolocumab (6.1 mg/kg/day) subcutaneously. Group V received evolocumab (6.1 mg/kg/day) and atorvastatin (3.5 mg/kg/day) orally.

The study had a duration of 10 weeks. Approximately 3 ml of blood was collected from the central ear vein of each rabbit following overnight fasting. Blood samples were collected at three-time points: baseline, 5 weeks, and 10 weeks for subsequent analysis. Serum was obtained from the blood samples and used for measuring inflammatory readouts. In the 10th week, the animals were anesthetized using ketamine and xylazine at a dosage of 5-10 mg/kg. Segments of the aorta were dissected from the rabbits for the measurement of intimal thickness and histopathological examination.

### Inflammatory biomarkers

Serum levels of interleukin (IL)-10, IL-17, IL-1β, intracellular adhesion molecule (ICAM), and vascular adhesion molecule (VCAM) were measured using an enzyme-linked immunosorbent assay (ELISA) kit.

### Lipid profiles test

After overnight fasting, blood samples were collected from the central ear vein and centrifuged. The supernatants were used to measure serum levels of total cholesterol (TC), triglycerides (TG), high-density lipoprotein-cholesterol (HDL-C), and low-density lipoprotein-cholesterol (LDL-C). Blood samples were collected at baseline, 5 weeks, and 10 weeks.

### Aorta intimal medial thickness

Intimal thickness measurements were performed using a light microscope.

### Histopathological procedure

Segments of the aorta were fixed in 10% formaldehyde and stained with hematoxylin-eosin for histopathological examination.

### Statistical analysis

Intimal thickness measurements were performed using a light microscope.

### Histopathological procedure

Data analysis was conducted using SPSS software version 21. All data were presented as mean ± standard deviation (SD) unless otherwise stated. The mean values among the study groups at different time points were compared using the t-test. A p-value of ≤0.05 was considered statistically significant.

## RESULTS

### Evolocumab decreased lipid profiles in atherosclerotic rabbits

In the high-fat diet group (Group II), serum levels of LDL-C, TC, and TG significantly increased after 10 weeks. However, treatment with evolocumab resulted in a marked reduction in LDL-C, TC, and TG (Table 1). Evolocumab was more effective in reducing the serum levels of TG, TC, and LDL-C compared to atorvastatin.

**Table 1.** Mean values of study parameters among the five study groups (N=10)

Group	Parameter	Baseline	5 weeks	10 weeks
Group I (Control)	TC mg/dl	59.86±15.16	61.56±19.00	61.19±14
	TG mg/dl	48.48±15.85	47±2.0	46±15.0
	LDL mg/dl	29±14.50	28±11.0	28.0±11.0
	HDL mg/dl	16.0±1.2	15.1.5±3.4	16.0±1.5
	IT μm	103.46±13.85		
Group II (Atherogenic diet)	TC mg/dl	63.94±16.15	717.64±209*	1301±443*
	TG mg/dl	42.05±3.51	196.4±45.35*	256.0±24.0*
	LDL mg/dl	30.0±11.0	576.0±190*	929±251.0*
	HDL mg/dl	18.0±4.2	16.0±3.5	16.0±2.1
	IT μm	248.43*±11.11		
Group III (DMSO)	TC mg/dl	63.52±13.17	785.98±271.00*	1136±371*
	TG mg/dl	47.25±16.68	187.4±26.15*	239.0±24*
	LDL mg/dl	24.0±11	460.0±75.0*	756.0±129.0*
	HDL mg/dl	18.0±3.2	17.0±2.5	15.0±3.0
	IT μm	214.17*±12.89		
Group IV (Evolocumab)	TC mg/dl	59.93±18.63	442.00±63*	291±50*α
	TG mg/dl	187.0±41.25*	97±18*α	41.01±5.81
	LDL mg/dl	33.0±8.0	486±42*	283.0±36*α
	HDL mg/dl	17.0±2.0	16.00±1.3	18.0±1.6
	IT μm	9.45±160.66		
Group V (Evolocumab & Atorvastatin)	TC mg/dl	61.30±16.50	374.00±95*	190±38α*
	TG mg/dl	148.0±22*	70.0±10* α	48.18±15.27
	LDL mg/dl	35.00±9	429.0±48*	209.0±33*α
	HDL mg/dl	17.0±2.8	18.0±2.6	26.00±4.2
	IT μm	121.79±5.3α*		

*p<0.05: there was a significant difference between groups III, IV, V, and group II in the study parameters at different time points. αp<0.05: there was a significant difference between group IV and group V in the study parameters at different time points. Data are presented as mean ± SD

### Evolocumab attenuated inflammation in atherosclerotic rabbits

The administration of a high-cholesterol diet for five weeks led to a significant increase in the serum levels of IL-17 (3.4±0.4 to 7.7±1) and IL-1β (1.04±0.19 to 9.66±1.4) at 10 weeks (Table 2). Additionally, ICAM and VCAM levels significantly increased from 1.7±0.15 to 8.2±0.74 and from 0.89±0.07 to 5.2±0.6, respectively (p<0.01). However, the co-administration of evolocumab and atorvastatin effectively reduced these increments. Furthermore, after 10 weeks of evolocumab treatment, there was a significant reduction in IL-1β levels from 5.13±0.73 to 2.66±0.36 (p<0.05). Similarly, the levels of ICAM decreased from 4.3±0.18 to 2.7±0.32 (p<0.05), and VCAM levels decreased from 3.8±0.34 to 1.6±0.15 (p<0.05). Notably, evolocumab significantly reduced the expression of TLR4 protein compared to the control group values (25.10±5.0 vs. 18.01±1.7 and 26.17±2.6 vs. 15.38±1.8) (p<0.05). The intimal thickness in the evolocumab-treated group decreased significantly (p<0.05) from 248.43±11.11 to 160.66±11.11 after 10 weeks of treatment. The combination group (Group V) demonstrated a significant reduction in lipid profile at both 5 and 10 weeks of treatment, with the most pronounced effect observed at 10 weeks compared to the high cholesterol diet group (Group II). Specifically, the combination treatment resulted in a decrease in total cholesterol from 374±95 to 190±38, triglycerides from 148.0±22.0 to 70.0±10, and LDL from 429±48 to 209±37 at 10 weeks. Additionally, the serum levels of IL-1β decreased from 5.17±0.67 to 2.18±0.31 at 10 weeks, while ICAM levels decreased from 4.4±0.25 to 2.4±0.32 and VCAM levels decreased from 4.20±0.35 to 1.5±0.19.

**Table 2.** Changes of various inflammatory parameters in evolocumab and evolocumab plus atorvastatin treated groups (N=10)

Group	Inflammatory marker	Period (Wk.)	No. animals	Mean
Group IV (evolocumab)	IL-1β	0	10	0.96±0.14
		5	10	5.13±0.73
		10	10	2.66±0.36*
	IL-17	0	10	3.38±0.13
		5	10	6.0±0.28
		10	10	5.20±0.32
	ICAM	0	10	1.80±0.05
		5	10	4.3±0.18
		10	10	2.70 ± 0.16*
	VCAM	0	10	0.90 ± 0.07
		5	10	3.80 ± 0.34
		10	10	1.60 ± 0.15*
Group II (atherogenic group)	IL-1β	0	10	1.04 ± 0.19
		5	10	5.5 ± 0.9
		10	10	9.66 ±1.4
	IL-17	0	10	3.40±0.40
		5	10	6.0±0.79
		10	10	7.70±1.00
	ICAM	0	10	1.70±0.16
		5	10	4.2±0.44
		10	10	8.20±0.74
	VCAM	0	10	0.89±0.07
		5	10	3.0±0.27
		10	10	5.20±0.45
Group V (evolocumab and atorvastatin)	IL-1β	0	10	0.98±0.13
		5	10	5.17±0.67
		10	10	2.18±0.31*
	IL-17	0	10	3.30±0.20
		5	10	5.90±0.31
		10	10	3.60±0.34*
	ICAM	0	10	1.80±0.07
		5	10	4.4±0.25
		10	10	2.40±0.32*
	VCAM	0	10	0.92±0.06
		5	10	4.0±0.35
		10	10	1.50±0.19*

*p<0.05: there was a significant difference between the atherogenic group and evolocumab, and atherogenic group and combination group (evolocumab and atorvastatin); Data are expressed as mean ± SD

TLRs count decreased from 25.79±6.5 to 11.65±2, which was statistically significant (p<0.01). Intimal thickness significantly decreased in the combination group at 10 weeks from 248.43±11.11 to 121.79±5.3 (p<0.01). In this study, all rabbits that were fed with an atherogenic diet developed varying stages of hypercholesterolemia and atherosclerosis lesions, including fatty streaks, atheroma, and fibrous cap formation in the branches of the aorta artery. Treatment with the combination of atorvastatin and evolocumab demonstrated a significant (p<0.01) reduction in the severity of atherosclerotic lesions compared to the untreated group (Table 1).

**Figure 1.**
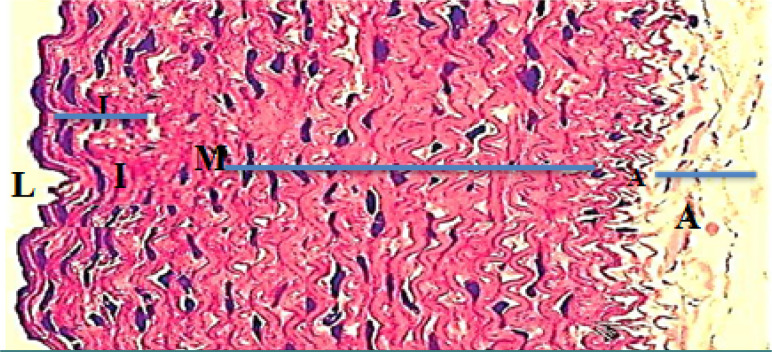
Histological section of the rabbit aorta in the control group showed normal layers represented by (L) Lumen (I) intima layer, (M), media layer, and (A) adventitia layer (H and E 400X)

**Figure 2.**
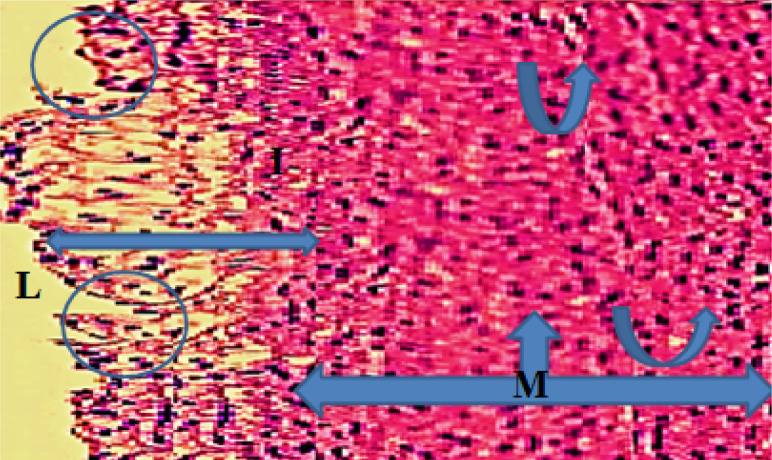
Histopathological section of the high cholesterol diet group showing aorta of rabbits with hypercholesterolemia (two-headed arrow) intima layer, (arrow) foam cells, (three-headed arrow) media layer, (arc arrow) cytoplasmic vacuolation, (circle) irregular surface (H&E, 100X magnification)

**Figure 3.**
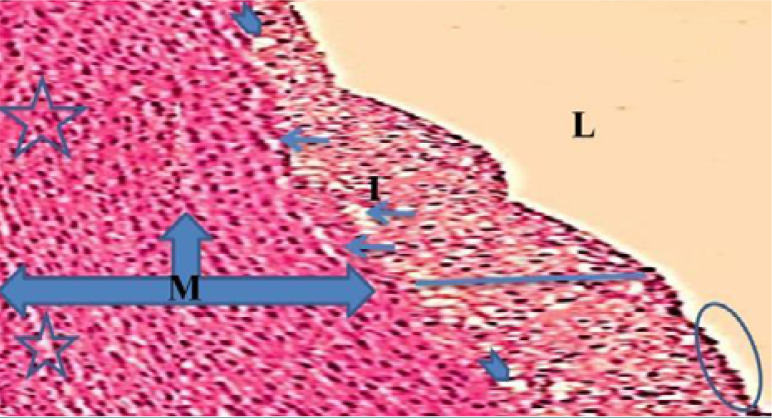
Histopathological section of the DMSO group showing rabbit aorta: (three-headed arrow) media layer, (arrow) fatty streak, (straight arrow) intima layer, (stars) cytoplasmic vacuolation, (head arrow) extracellular lipid pool, (circle) slight hemorrhages of intima surface (H&E, 100X magnification)

**Figure 4.**
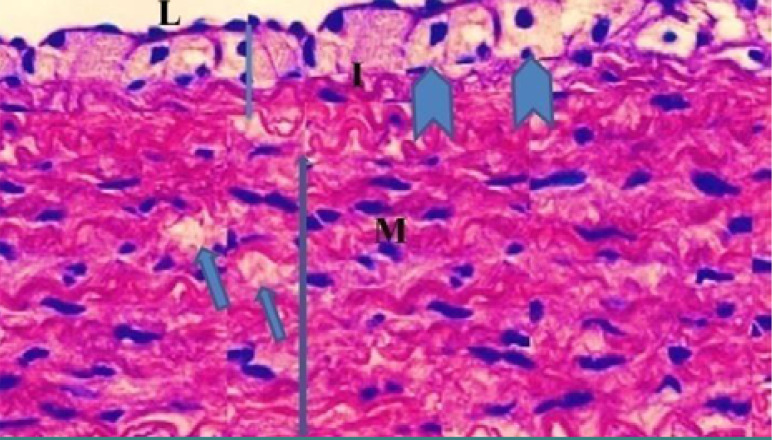
Histopathological section of the evolocumab group showing rabbit aorta: (straight I) intima layer, (thick arrow) fatty streak, (two-headed arrow M) media layer, and (arrow) foam cell (H&E, 400X magnification)

**Figure 5.**
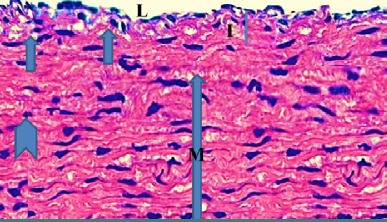
Histopathological section of rabbit aorta in the atorvastatin plus evolocumab group showed (straight I) normal intima layer (two head arrow M) normal media layer, (arrow) slight layer of foam cell and (square) fiber degeneration (H&E, 400 X magnification)

Monocyte expression of Toll-like receptors 2 and 4 was significantly lower (p<0.05) in the evolocumab group compared to the atherogenic group at 5-10 weeks (Table 3). The control group showed normal histological layers of the aorta (Figure 1), the high-cholesterol diet group presented hypercholesterolemia (Figure 2), the DMSO group showed fatty streak in the intima layer, cytoplasmic vacuolation, extracellular lipid pool lipid, and slightly hemorrhages of intima surface (Hematoxylin and Eosin (H&E) staining, 100X magnification) (Figure 3). Moreover, the evolocumab group presented fatty streaks and foam cells (H&E, 400 X magnification) (Figure 4). The fifth group, the Atorvastatin plus Evolocumab group, showed a normal intima layer, normal media layer, a slight layer of foam cell, and fiber degeneration (H&E, 400 X magnification) (Figure 5).

**Table 3.** Effect of evolocumab alone and in combination with atorvastatin on peripheral blood monocyte expression of TLR2 and TLR4 at 5 and 10 weeks (N=10)

TLR2%			TLR4%			
Baseline	5 weeks	10 weeks	Baseline	5 weeks	10 weeks	
Group V	25.79±6.50	16.63±1.20	11.65±2α	24.09±3.90	5.91±0.64α#	5.08±1.20α#
Group IV	25.79±6.50	18.01±1.70	15.38±1.80α	24.09±3.90	11.83±1.20α	10.83±1.80α
Group II	25.4±4.41	25.1±5.0	26.17±2.6	24.6±2.5	24.30±4.30	25.10±4.10

#p<0.05: There was a significant difference between group IV and V at different time points αp<0.05: There was a significant difference in the group II versus the group IV, group V at different time points

## DISCUSSION

The present study demonstrates the beneficial effects of Evolocumab, alone or in combination with atorvastatin, in reducing the deterioration of atherosclerotic plaque. The high-cholesterol diet led to a significant increase in all lipid profile parameters, which is consistent with previous studies [13]. The rabbits in this study rapidly developed hypercholesterolemia, with serum cholesterol levels exceeding 1000mg/dl following oral cholesterol feeding. Within 10 weeks, atherosclerotic changes in the form of foam cells were observed. It is worth noting that a longer treatment period was not feasible due to associated failure to thrive and liver toxicity concerns [14]. Rabbits are particularly sensitive to dietary cholesterol due to their limited ability to increase sterol excretion, resulting in increased LDL levels, which play a crucial role in the formation of atherosclerotic plaques [15].

Statins are a family of effective therapeutic agents that are commonly used in reducing levels of lipids in circulation. Other medications, such as PCSK9 inhibitors, are found to be critical players by interfering with the process of LDL receptors reaching the plasma membrane of hepatocytes after receptor internalization, thereby limiting the removal of LDL particles from circulation [16]. Evolocumab is a monoclonal antibody targeting PCSK9. It has been reported that treatment with Evolocumab or a combination of both Evolocumab and statins resulted in a marked reduction in levels of LDL-C [17]. In this study, treatment with Evolocumab, either alone or in combination with atorvastatin, resulted in a significant reduction in TC, LDL-C, and TG levels, with evolocumab showing greater efficacy compared to atorvastatin, consistent with previous findings.

IL-1β, a pleiotropic cytokine implicated in the pathogenesis of atherosclerosis, was found to be significantly elevated in the atherogenic group compared to the control group. IL-1β is known to induce the expression of adhesion molecules such as ICAM and VCAM in vascular smooth muscles, thereby promoting atherosclerosis progression [18]. The high cholesterol diet led to a significant increase in ICAM and VCAM levels, which are immunoglobulin-like adhesion molecules involved in the interaction between endothelial cells and blood cells, further contributing to atherosclerosis development [19,20]. Recently it was found that VCAM is mainly up-regulated following a high-cholesterol diet [21].

Hypercholesterolemia can result in the augmentation of TLRs signaling, possibly leading to the promotion of inflammatory responses [22]. There is growing evidence of the role of TLR in the initiation and progression of atherosclerosis [13]. Edfeldt K. *et al*. (2002) demonstrated that TLR2 and TLR2 expression is significantly elevated in endothelial cells overlying atheroma, similar to our findings [23]. The expression of TLR2 and TLR4 at the wall of the blood vessel can enhance atherosclerosis in a synergistic way [24]. Mullick *et al*. showed that in atherosclerosis-susceptible low-density lipoprotein receptor-deficient mice, TLR2 knockout mice led to a reduction in atherosclerosis, results that are in line with current study findings.

The present study demonstrated that evolocumab effectively reduced the size of atherosclerotic lesions, and the addition of atorvastatin further enhanced these effects. These findings align with previous research indicating that evolocumab reduces monocyte recruitment and modulates the composition of lesions, resulting in a more stable plaque morphology characterized by increased collagen and smooth muscle cell content, as well as decreased necrotic core and macrophage presence [25].

Atherosclerotic plaques consist of distinct areas, including necrotic and macrophage-rich regions associated with inflammatory processes, as well as the fibrotic cap composed of collagen and smooth muscle cells, representing protective factors [26]. In this study, both evolocumab alone and the combination of evolocumab with atorvastatin significantly reduced inflammatory factors compared to the control group. Additionally, the combination treatment demonstrated an increase in protective factors when compared to the control group.

## CONCLUSION

The treatment with evolocumab resulted in a statistically significant reduction in lipid levels at 5 weeks, with the most pronounced effects observed at 10 weeks. Additionally, it effectively lowered TLR expression (10.83±1.8) and intimal thickness (160.66±9.45) at 10 weeks. IL-17, IL-1β, ICAM, and VCAM were significantly reduced by evolocumab treatment, with the improvement of the histopathological changes in the aortic wall. The combination of evolocumab plus atorvastatin caused a more statistically significant reduction in TLRs at 10 weeks (5.08 ±1.2) and intimal thickness (121.79±5.3). These findings emphasize the potential of evolocumab as a therapeutic agent for delaying the advancement of atherosclerosis by modulating inflammatory pathways.. Further research is required to establish the therapeutic efficacy of evolocumab in terms of atherosclerosis.
